# Somatosensory and Pharyngolaryngeal Auras in Temporal Lobe Epilepsy Surgeries

**DOI:** 10.1155/2013/148519

**Published:** 2013-06-03

**Authors:** Alexander G. Weil, Werner Surbeck, Ralph Rahme, Alain Bouthillier, Adil Harroud, Dang Khoa Nguyen

**Affiliations:** ^1^Division of Neurosurgery, Notre Dame Hospital, University of Montreal Medical Center (CHUM), Montreal, QC, Canada; ^2^Division of Neurology, Notre Dame Hospital, University of Montreal Medical Center (CHUM), 1560 Rue Sherbrooke Est, Montreal, QC, Canada H2L 4M1

## Abstract

*Purpose*. Somatosensory (SSA) and pharyngolaryngeal auras (PLA) may suggest an extratemporal onset (e.g., insula, second somatosensory area). We sought to determine the prognostic significance of SSA and PLA in temporal lobe epilepsy (TLE) patients undergoing epilepsy surgery. *Methods*. Retrospective review of all patients operated for refractory TLE at our institution between January 1980 and July 2007 comparing outcome between patients with SSA/PLA to those without. *Results*. 158 patients underwent surgery for pharmacoresistant TLE in our institution. Eleven (7%) experienced SSA/PLA as part of their seizures. All but one had lesional (including hippocampal atrophy/sclerosis) TLE. Compared to patients without SSA or PLA, these patients were older (*P* = 0.049), had a higher prevalence of early ictal motor symptoms (*P* = 0.022) and prior CNS infection (*P* = 0.022), and were less likely to have a localizing SPECT study (*P* = 0.025). A favorable outcome was achieved in 81.8% of patients with SSA and/or PLA and 90.4% of those without SSA or PLA (*P* > 0.05). *Conclusion*. Most patients with pharmacoresistant lesional TLE appear to have a favorable outcome following temporal lobectomy, even in the presence of SSA and PLA.

## 1. Introduction

Recent evidence suggests that failure to recognize insular cortex seizures could be responsible for some cases of surgical failure in patients with temporal lobe epilepsy (TLE) [1–5]. Insular seizures may mimic TLE or may coexist with temporal seizures, an entity referred to as temporal plus epilepsy [6–8]. Clinical observation of patients with insular seizures proven by depth recordings and after cortical stimulation using insular contacts has revealed a high prevalence of somatosensory and pharyngolaryngeal auras (SSA and PLA, resp.), including a characteristic sensation of laryngeal constriction (LC) [1–3, 9–15]. In this study, we sought to determine the prevalence and prognostic significance of SSA and PLA in TLE patients undergoing epilepsy surgery.

## 2. Materials and Methods

We performed a retrospective chart review of all patients who underwent surgery for refractory TLE at our institution between January 1980 and July 2007. All patients underwent a comprehensive epilepsy surgical workup, including complete anamnesis and neurological examination, neuropsychological evaluation, and video-electroencephalographic (VEEG) monitoring with scalp electrodes. Magnetic resonance imaging (MRI) were performed in all patients after 1992. Single photon emission computed tomography (SPECT) and ^18^F fluorodeoxyglucose positron emission tomography (PET) was performed in more recent cases. Invasive VEEG recordings were obtained in the majority of patients before the availability of MRI. Since then, electrode implantation has been performed only in well-selected cases following evaluation by our epilepsy multidisciplinary team.

Collected data included patient demographics, cause of epilepsy and risk factors, seizure type and clinical features, presence and characteristics of SSA and/or PLA, number of antiepileptic drugs tried, type of resective surgery, histopathological findings, and final outcome in seizure control. SSAs were defined as a perceptual experience of tingling, numbness, electric-shock sensation or pain, occurring in isolation as a simple partial seizure or as an early manifestation (i.e., aura) of a complex partial seizure [16–18]. PLAs were defined as ictal pharyngolaryngeal symptoms of paresthesia (tingling or burning), discomfort, or sensation of throat constriction of varying intensity [1–3, 19–22]. Other early ictal symptoms suggestive of operculoinsular involvement were also documented, including motor, epigastric viscerosensory, cephalic, auditory, and dysphasic symptoms. Outcome was classified using the Engel classification system [23]. A favorable outcome was defined as Engel I or II. The study protocol was approved by the institutional review board.

We compared subgroups of patients with SSA/PLA to those without SSA/PLA. Categorical (binomial) variables were compared using Boschloo exact unconditional test and continuous variables using the Wilcoxon rank sum test. Statistical analysis was performed using R [24, 25] and PASW Statistics 18 (SPSS Inc., Chicago, IL, USA). A *P* value <0.05 was considered to be statistically significant.

## 3. Results

### 3.1. Study Population and Patient Characteristics

During the study period, a total of 158 patients underwent surgery for pharmacoresistant TLE in our institution. This patient population consisted of 74 women (47%) and 84 men (53%) with an average age of 33.9 years (range 13–62) and an average duration of epilepsy of 21 years (range 1–46). Risk factors for epilepsy included CNS infection in 20 (12.7%), head trauma in 12 (5%), positive family history for epilepsy in 33 (21%), febrile seizures in 34 (22%), perinatal complications in 10 (6%), and developmental delay in 10 (6%). MRI was obtained in 114 (72%) of these patients, revealing abnormalities in most cases (87%), hippocampal atrophy (HA) without sclerosis in 22 (19%), hippocampal sclerosis (HS) in 54 (47%) and other temporal lobe abnormalities in 51 patients (32%). Ictal scalp EEG suggested unilateral temporal lobe origin in 88 (58%) cases. Intracranial electrodes were implanted in 76 patients (48%). Sixty-five (67%) ictal SPECT scans localized the seizure focus to one temporal lobe. FDG PET was only obtained in 15 patients, and it localized the seizure focus to the temporal lobe in 13 of these patients (87%). Patients had tried 4.4 AEDs (range 2–12) on average prior to surgery. Twenty-seven underwent selective amygdalo-hippocampectomy (SAH) (17%), 75% underwent anterior medial temporal lobectomy (AMTL) (*n* = 118), 4% AMTL and lesionectomy (*n* = 6), and 4% lesionectomy (*n* = 7). AMTL involved resection of 3.5–7 cm T2, T3, most (two-thirds) of the amygdala, and a radical hippocampectomy. Pathology confirmed HS in 69 (44%), HS and another pathology in 9 (5.7%), ganglioglioma in 6 (3.8%), glioma in 5 (3.2%), cavernoma in 4 (3%), focal cortical dysplasia in 4 (3%), gliosis in 3 (2%), dysembryoplastic neuroepithelial tumor in 2 (1%), was normal in 24 (15%), and was inconclusive in 16 (10%).

### 3.2. Subgroup Analysis: Patients with SSA and/or PLA

*A-General Characteristics. *Of all patients, only 11 (7%) experienced symptoms of SSA and/or PLA as part of their seizures: SSA (*n* = 8, 5%), PLA (*n* = 2, 1.2%), or both (*n* = 1, 0.6%) (Table 1). This group of patients was composed of 3 men and 8 women with mean epilepsy duration of 27 years (range 2–46) and mean age at surgery of 40 years (range 21–55). Risk factors for epilepsy included central nervous system (CNS) infections in 4 patients, perinatal anoxia in 1, febrile seizures in 1, and family history of epilepsy in 1 (Table 2). No risk factors were identified in 4 patients. MRI was obtained in 10 patients, revealing HS in 6, HA in 2, a posterior temporal cavernous malformation in 1, and a fusiform gyrus pilocytic astrocytoma in 1. Scalp EEG recordings localized the seizure focus to the temporal lobe in 7 patients (64%), lateralized the focus to the hemisphere ipsilateral to the resection side in 3 (27%), and were non-localizing in 1 (9%) patient. Seven patients underwent preoperative ictal SPECT, which localized the seizure focus to the temporal lobe in 2 and was nonlocalizing in 5 cases. Intracranial electrodes were implanted in 6 patients, enabling localization of seizure focus to the mesial temporal lobe in all of them. Patients were on an average of 4.8 antiepileptic medications (range 2–9). Surgical procedures included AMTL in 7 patients, SAH in 2, lesionectomy and temporal corticectomy in 1, and simple lesionectomy in 1. Three patients underwent a second operation during the study period (before outcome assessment). One patient with previous cavernoma lesionectomy and corticectomy underwent second operation with electrocorticography (ECoG) guided further perilesional corticectomy. Two patients with initial SAH underwent a second resective surgery consisting of radical temporal lobectomy, including the superior temporal gyrus. Pathology demonstrated HS either alone (*n* = 7) or with another pathology (*n* = 1) in 8 patients, a cavernous malformation in 1, a pilocytic astrocytoma in 1, and was inconclusive in 1.

 Compared to patients without SSA or PLA, those with SSA and/or PLA were older at surgery (*P* = 0.049), had a higher prevalence of early ictal motor symptoms (*P* = 0.022), had a higher rate of CNS infections (*P* = 0.022), and were less likely to have a localizing SPECT study (*P* = 0.025) (Table 3).

*B-Seizure Characteristics. *Eight patients had only SSA, 2 had only PLA, and 1 had both SSA and PLA. Of the 9 SSA patients, 7 (77.8%) had strictly unilateral symptoms involving the contralateral hemiface in 2, the contralateral hand in 2, the contralateral hemibody in 1, the ipsilateral leg in 1, and the ipsilateral hemibody in 1. In 1 patient with contralateral hemiface SSA, symptoms progressed in a somatotopic Jacksonian march to the ipsilateral hemitongue, arm, and leg. In 2 patients (22.2%), SSAs were strictly bilateral, involving the legs in 1 and the forearms in the other. In the 3 patients with PLA, symptoms were described as a sensation of pharyngo-laryngeal constriction (*n* = 3), with occasional pharyngeal pain in 1 patient.

*C-Surgical Outcomes. *Outcomes were assessed in all but 1 patient who was lost to followup in the no SSA/PLA group (*n* = 157). The mean followup was 7.2 years. A favorable outcome (Engel I or II) was achieved in 81.8% (9/11) of patients with SSA and/or PLA and 90.4% (132/146) of those without SSA or PLA (*P* > 0.05) (Table 4). A complete seizure-free outcome (Engel Ia) was observed in 36.4% (4/11) of patients with SSA and/or PLA and 32.2% (47/146) of those without SSA or PVA (*P* > 0.05).

Since the study period, two patients of the SSA/PLA cohort have undergone invasive reinvestigation for persistent disabling seizures (Table 2). Both have obtained seizure-freedom following a third resection of the temporal lobe and anterior insular cortex, respectively.

## 4. Discussion

Insular epilepsy should be suspected whenever TLE-like seizures are accompanied by early-occurring LC and/or somatosensory symptoms (SSS), especially those described as unpleasant paresthesias or warmth affecting the perioral region or extending to a large somatic territory, either bilateral or ipsilateral to the seizure focus [1–3, 5]. Although some studies have suggested that patients with pharmacoresistant TLE who exhibit SSA and PLA have a favorable prognosis following temporal lobe surgery [8, 21, 26, 27], others have reported a poor outcome in this patient population [28–30]. The purpose of this study was to determine the prevalence and prognostic significance of SSA/PLA as a potential marker of insular epilepsy and thus a predictor of poor response to surgery in TLE patients.

### 4.1. Prevalence of SSA and PLA in Surgical TLE Patients

The 5% rate of SSA among surgical TLE patients in the present series is comparable to the 1.7%–14% rates reported in the literature [26, 29, 31–35]. Unlike prospective studies [26] that have reported an 11% incidence of SSA in TLE surgery patients, retrospective series may actually underestimate the real prevalence of SSA, mainly as a result of recall bias. Although viscerosensory auras are frequent (45%) in patients with pharmacoresistant TLE [33], the prevalence of PLA is less well known. Pharyngeal auras have been reported to occur in up to 16.9% of pharmacoresistant TLE and 13% of temporal plus epilepsy cases [8]. The higher prevalence in the series of Barba et al. compared to ours may either reflect a difference in the definition of PLA or a selection bias since all patients in their study had undergone intracranial electrode implantation [8].

### 4.2. Failure of SSA and PLA to Predict Worse Surgical Outcomes: Significance and Implications

In this study, SSA and/or PLA were not found to be negative prognostic factors in TLE patients undergoing surgery. Our findings are in line with previous reports suggesting that SSA [8, 26, 27] and PLA [21, 22] do not necessarily indicate an extratemporal seizure onset or an independent extratemporal seizure focus in patients with pharmacoresistant temporal lobe-like epilepsy. Our findings would suggest that for the majority of our patients, SSA or PLA was the result of rapid spread of epileptic activity to perisylvian somatosensory structures such as the insular cortex and second somatosensory cortex (SII) [1, 2, 5, 6, 22, 26, 35–37] rather than from an extratemporal seizure focus.

Because almost all of the patients in the SSA/PLA cohort had lesional TLE, it is not possible to draw any conclusion about the prognosis of SSA/PLA in nonlesional TLE surgery patients. For the latter, it would remain prudent to perform an intracranial study to rule out an extratemporal focus. For the former, however, temporal lobe surgery without preoperative invasive investigation would appear to result in a good outcome for most. Although independent insular seizures have been known to coexist with temporal lobe seizures and HA/HS (i.e., the hippocampal MRI abnormality may represent only the tip of the iceberg of a larger pathological substrate), it may be that this is a rare occurrence which does not necessarily warrant systematic invasive investigation in the presence of SSA/PLA. In our series, this situation was encountered in only one subject (patient 3). Isnard et al. [2] sampled the insula in 50 consecutive patients with TLE on the basis of ictal symptoms or scalp VEEG data suggesting an early spread of seizures either to the suprasylvian opercular cortex (e.g., lip and face paresthesiae, tonic-clonic movements of the face, dysarthria, motor aphasia, gustatory illusions, hypersalivation, and postictal facial paresis) or the infrasylvian opercular cortex (e.g., auditory hallucinations, early sensory aphasia). Only five patients (10%) had seizures originating from the insular cortex while in 43 patients (86%), they propagated to the insula after a temporal onset. Hopefully, further clinical observations and imaging techniques (e.g., magnetoencephalography) will allow us to better identify the small subset of patients who will most benefit from an implantation prior to surgery [38, 39].

### 4.3. Study Limitations

The retrospective nature of this study may be associated with a recall bias for the incidence and characteristics of SSA/PLA. These auras are very subjective and are very much dependent on the history taking skills and detailed attention of the examiner. Furthermore, because they were not assessed in a standardized way, the timing of these auras during a seizure may not always be clear with regards to other subjective symptoms patients may have felt at seizure onset. Whether SSA/PLA occurs as the initial or only aura may be important [28, 29]. Many of the patients in this report had multiple auras, some more typical of mesial temporal lobe epilepsy (déjà vu, epigastric). Finally, as mentioned previously, our data does not allow drawing conclusions about the prognosis of SSA/PLA in non-lesional temporal lobe-like epilepsy as numbers are too small. It is possible that these patients have been selected out already by careful presurgical evaluation and found to have extratemporal or temporal plus epilepsy.

## 5. Conclusion

Most patients with pharmacoresistant lesional TLE appear to have a favorable outcome following temporal lobectomy, even in the presence of SSA and PLA.

## Figures and Tables

**Table 1:** Demographics and clinical features in 11 patients with SSA and/or PVA.

Pt	Sex, age (yr)	Duration of epilepsy (yr)	Seizure characteristics	
			Aura	Late manifestations
1	F, 47	24	L hand SS, cephalic	L hand dystonic, R versive, oroalimentary automatisms, L versive, gestural automatisms
2	F, 21	2	bilateral forearm SS	oroalimentary and manual automatisms
3	M, 46	26	olfactory, gustatory, epigastric VS, hypersalivation, R palpebral myoclonic, R leg SS, mnemonic déja vu	oroalimentary and manual automatism, affective (psychomotor agitation)
4	F, 55	15	L hemibody SS, epigastric VS, face myoclonic,	R versive, oroalimentary automatism, L hand dystonic
5	F, 36	35	R hand SS, LC, R arm SM	R arm dystonic, R arm, and R hemiface myoclonic
6	F, 47	27	2 types: 1, L hemiface then L hemitongue, then L arm, then L leg SS, cephalic; 2:affective (fear)	type 2: affective (fear), L arm manual and oroalimentary automatisms, verbal (jargon) automatisms, thrusting, then L motor tonic L versive then L hemiface myoclonic
7	M, 39	38	2 types: 1, LC and laryngeal pain; 2, epigastric VS	LOC, oroalimentary automatisms, dyspraxia
8	F, 43	37	LC, epigastric VS, then mnesic (déjà vu, deja vecu), generalized warmth	2 types, 1, aura only; 2, LOC, gestural automatisms
9	F, 34	17	2 types: 1, epigastric VS, olfactory, déjà vu; 2, R hemiface SS	oroalimentary automatisms, manual automatisms, vocal, R face myoclonic, generalization
10	F, 26	19	2 types: 1, epigastric VS, affective; 2, bilateral leg SS	auditory (remote noise/vice), LOC, oroalimentary automatisms, manual automatisms, hypersalivation, verbal
11	M, 48	46	2 types: 1, dysgeusia, affective; 2, L hemibody SS	LOC, oroalimentary automatisms, hypermotor agitation, R versive, R arm dystonia, generalization

Pt: patient; M: male; F: female; yr: years; L: left; R: right; SS: somatosensory; LC: laryngeal constriction; SM: somatomotor; LOC: loss of contact; VS: viscerosensory.

**Table 2:** Findings of presurgical work-up, surgical procedures performed, pathology results, and final outcomes in 11 patients with SSA and/or PVA.

Pt	Risk factor	MRI	Ictal SPECT	sEEG	Intracranial electrode study			First surgery	Second surgery	FU (yrs)	Outcome	Re-investigation	Third surgery	Final outcome
					Performed (y/n)	Coverage	Focus							
1	None	R PT Cavernoma	NL	Loc	N	—	—	R cavernoma resection, corticectomy	ECoG, perilesional corticectomy	4	IIIa	R post T, R T et lat inf, R H, R I	R ATL	Ia
2	Fam	L T pilocytic astrocytoma	NL	Loc	N	—	—	L tumor resection	—	1	Ia (Rx)	—	—	—
3	None	R HS	—	Loc	N	—	—	R SAH	R ATL including TO	1	IIIa	R I, residual TL, FPO, OF	R insulectomy	Ia
4	None	R HS	NL	Loc	Y	R F, T, Pd, A, H	R MT	R ATL	—	7	IIb		—	—
5	Encephalitis (6 months) residual R hemiparesis, mental retardation	L HS, L hemisph atrophia	L	Lat	Y	L H, L A, L subtemp ant post, L FT	L MT	L ATL	—	9	Ib		—	—
6	None	R HS	L	Loc	N	—	—	R ATL	—	8	Ia (Rx)	—	—	—
7	Viral encephalitis (8 months)	L HA	—	Loc	N	—	—	L ATL	—	13	Ia (No Rx)	—	—	—
8	Encephalitis (8 months)	R HA	NL	Loc	Y	R H, R A, L H	R MT	R ATL	—	13	IIc	—	—	—
9	Meningitis (12 months)	L F, L T atrophia, L T gliosis	—	NL	Y	NA	L MT	L ATL	—	4	Ib	—	—	—
10	None	—	—	Lat	Y	R A, R H, R PT, R cingular gyrus ant, R SMA, L H, L cingular gyrus ant	R MT	R ATL	—	10	Ia (No Rx)	—	—	—
11	None	L HS	NL	Lat	N	—	—	L SAH	L ATL (4 cm T1, 5 cm T2, 7 cm T3)	1	IIb	—	—	—

MRI: magnetic resonance imaging; sEEG: scalp EEG; Y: yes; N: no; SSA: somatosensory aura; F: frontal; T: temporal; P: parietal; O: occipital; A: amygdala; H: hippocampus; R: right; L: left; MT: mesial temporal lobe; FU: followup; Fam: family history of epilepsy; PT: posterior temporal; HS: hippocampal sclerosis; HA: hippocampal atrophy; Loc: localized; Lat: lateralized to hemisphere; SAH: selective amygdalohippocampectomy; ATL: anterior temporal lobectomy; ECoG: electrocorticography; TO: temporal operculum; TFG: temporal fusiform gyrus; I: insula; NL: nonlocalizing; hemisph: hemisphere; Rx: medication.

**Table 3:** Baseline characteristics of patients in the SSA/PVSA group and controls.

Variable	SSA/PVSA (*n* = 11)	Controls (*n* = 147)	*P*-value
Demographic			
Female sex	8 (72.7%)	66 (44.9%)	0.095
Age at surgery (yrs)	40.18	33.44	0.049
Duration of epilepsy (yrs)	26.91	20.84	0.086
Seizure characteristics			
Daily seizures or worse	2 (18.2%)	38 (25.9%)	0.649
Early ictal symptoms suggestive of insular involvement			
Epigastric symptoms	6 (54.5%)	54 (36.7%)	0.280
Motor symptoms	4 (36.4%)	16 (10.9%)	0.022
Auditory symptoms	0 (0%)	7 (4.8%)	1.000
Cephalic symptoms	1 (9.1%)	27 (18.4%)	0.572
Dysphasic symptoms	0 (0%)	7 (4.8%)	1.000
Generalized seizures	7 (63.6%)	94 (63.9%)	1.000
Status epilepticus	1 (9.1%)	6 (4.1%)	0.296
Number of AEDs tried	5.00	4.36 (*n* = 144)	0.280
Left side	5 (45.5%)	91 (61.9%)	0.292
Etiology			
History of CNS infection	4 (36.4%)	16 (10.9%)	0.022
History of head trauma	0 (0%)	12 (8.2%)	1.000
Family history	1 (9.1%)	32 (21.8%)	0.406
Febrile seizures	1 (9.1%)	33 (22.4%)	0.405
Perinatal complications	1 (9.1%)	9 (6.1%)	0.374
Developmental delay	1 (9.1%)	9 (6.1%)	0.374
imaging			
Normal MRI	0/10 (0%)	15/104 (14.4%)	0.301
Hippocampal atrophy	2/10 (20%)	20/104 (19.2%)	1.000
Hippocampal atrophy and sclerosis	6/10 (60%)	48/104 (46.2%)	0.466
Other temporal lesion	5 (45.5%)	46 (31.3%)	0.270
Scalp EEG			
Localizing to TL	7 (63.6%)	81/144 (56.3%)	0.690
Lateralizing	3 (27.3%)	34/144 (23.6%)	0.641
Nonlateralizing	1 (9.1%)	29/144 (20.1%)	0.615
SPECT localizing to TL	2/7 (28.6%)	63/89 (70.8%)	0.025
FDG PET			
Normal	0/0 (?)	2/15 (13.3%)	N/A
Temporal focus	0/0 (?)	13/15 (86.7%)	N/A
Intracranial electrode implantation	6 (54.5%)	70 (47.6%)	0.692
Surgery			
SAH	2 (18.2%)	25 (17%)	0.839
ATL	7 (63.6%)	111 (75.5%)	0.417
ATL and lesionectomy	0 (0%)	6 (4.1%)	1.000
Lesionectomy	2 (18.2%)	5 (3.4%)	0.039
ECOG	0 (0%)	4 (2.7%)	0.610
Pathology			
Sclerosis	7 (63.6%)	62 (42.2%)	0.179
Cavernoma	1 (9.1%)	3 (2%)	0.162
Glioma	1 (9.1%)	4 (2.7%)	0.199
Ganglioglioma	0 (0%)	6 (4.1%)	1.000
Focal cortical dysplasia	0 (0%)	4 (2.7%)	0.610
DNET	0 (0%)	2 (1.4%)	1.000
Gliosis	0 (0%)	3 (2%)	0.572
Normal	0 (0%)	24 (16.3%)	0.172

**Table 4:** Impact of somatosensory auras (SSA) and pharyngeal viscerosensory auras (PVSA) on surgical outcome.

SSA and/or PVSA	Surgical outcome		Total (*n*)
	Engel class I or II (*n*)	Engel class III or IV (*n*)	
Yes	9	2	11
No	132	14	146

	141	16	157

*P* = 0.244 (Boschloo unconditional exact test).
